# Intelligent Identification of Micro-NPR Bolt Shear Deformation Based on Modular Convolutional Neural Network

**DOI:** 10.3390/s26010184

**Published:** 2025-12-26

**Authors:** Guang Han, Chen Shang, Zhigang Tao, Xu Yang, Bowen Du, Xiaoyun Sun, Liang Geng

**Affiliations:** 1 Hebei Provincial Collaborative Innovation Center of Transportation Power Grid Intelligent Integration Technology and Equipment, Shijiazhuang Tiedao University, Shijiazhuang 050043, China; shangchen812702@163.com (C.S.); 18713832827@163.com (X.Y.); dubowen0311@163.com (B.D.); stdu_intbox@126.com (X.S.); 2 School of Electrical and Electronic Engineering, Shijiazhuang Tiedao University, Shijiazhuang 050043, China; 3 State Key Laboratory for GeoMechanics and Deep Underground Engineering, China University of Mining Technology, Beijing 100083, China; taozhigang1981@163.com; 4 North China Institute of Aerospace Engineering, Langfang 065099, China; 5 State Grid Hebei Electric Power Company, Ltd., Shijiazhuang 050023, China; maoer0059@163.com

**Keywords:** micro-NPR bolt, shear deformation identification, stress wave nondestructive detection technology, modular convolutional neural network

## Abstract

As an important means of reinforcement and support, the bolt can effectively resolve the problem of slope instability. Micro-Negative Poisson Ratio (Micro-NPR) bolts are superior to conventional bolts in mitigating large deformations caused by geological shifts. A large number of bolt anchoring systems require non-destructive testing technology for quality inspection. This technology utilizes time-domain signal characteristics to detect internal defects in the bolt anchoring systems of support engineering. The combination of stress wave nondestructive detection technology and modular convolutional neural network method can identify the shear deformation in the case of the anchor slope support. Integrating the identification results of both the shear angle and shear location sub-modules improves the accuracy of detecting shear deformation in micro-NPR bolt anchoring system, which will be of great assistance in our future engineering applications.

## 1. Introduction

**Figure 1:**
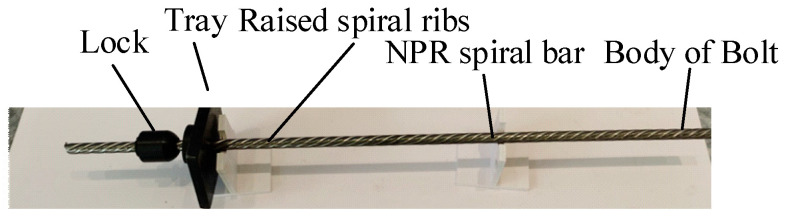
Physical structure diagram of the micro-NPR bolt.

Constant Resistance Energy Absorption (CREA) rock bolts effectively enhance slope mechanical properties by absorbing rock strain energy and accommodating large rock deformations. Micro-NPR bolts represent the most typical CREA bolts currently, as shown in Figure 1.

Compared to traditional bolts of equivalent cross-sectional area, micro-NPR bolts exhibit higher stiffness and superior shear resistance, featuring prominently raised helical ribs on their outer surface. These pronounced helical ribs increase the contact area between the bolt body and grout, thereby enhancing load-bearing capacity. In complex geological environments, traditional bolt support fails [1] due to its inability to adapt to the instantaneous large impact and continuous large deformation that occur after rock and soil deformation [2,3,4]. For deep and complex geological conditions, using micro-NPR bolts for support can reduce engineering geological hazards.

To further ensure the safety of the micro-NPR bolt anchoring system during support operations while saving costs associated with manual inspection and anchor replacement, shear deformation testing of micro-NPR bolts is crucial. The varying degrees of shear deformation in bolts caused by slope slip surface movement directly impact the safety of the bolt support system. Shear deformation and failure occur within the slope during bolt support operations, making direct visual observation impossible. Non-destructive testing technologies, with their advantages of intelligence, speed, and large-scale detection capabilities, can directly capture the acoustic time-domain signals generated when bolts undergo shear deformation at the slip surface. Micro-NPR bolts exhibit non-magnetic and corrosion-resistant properties, making stress wave non-destructive testing an ideal method for detecting shear deformation in their anchoring systems. Although stress wave testing has been applied to anchor bolt health monitoring, existing methods struggle to simultaneously and accurately analyze both the angle and location information of shear deformation.

There are two primary contributions of this paper:

(1) This paper proposes for the first time an intelligent identification framework that combines stress wave time-domain waveform acquisition with a dual-module convolutional neural network. The proposed method directly utilizes time-domain signal features without requiring prior feature extraction from acquired signals, enabling convenient and rapid identification and detection.
(2) This method can directly perform identification and detection based on time-domain signal waveforms, eliminating the need for preliminary feature extraction from the acquired signals, thereby enabling a convenient and efficient identification process.

## 2. Related Works

Through anchor shear tests, Tao et al. [1] discovered that the shear strength of micro-NPR bolts significantly exceeds that of threaded steel bolts, thus demonstrating the micro-NPR bolt shaft material’s capacity for large-deformation energy absorption. He et al. [5] further investigated the shear performance of micro-NPR bolts, conducting laboratory shear tests on bolt nodes under various normal stress conditions. Wu et al. [6] conducted a series of single shear tests to investigate the shear resistance of fully enclosed rock bolts and energy-absorbing rock bolts embedded in coarse joints under normal loading conditions. In the same conditions, the energy-absorbing bolts exhibited greater ultimate shear displacement. Yin et al. [7] developed a more precise and complex resilience model to provide cost-effective post-earthquake repair systems. They investigated key factors influencing the mechanical performance of shear bolts and predicted the post-buckling strength and collapse load of the node model.

Traditional bolt quality inspection methods in engineering [7] suffer from limited monitoring scope and the risk of damaging the anchoring system, whereas non-destructive testing technologies offer advantages such as intelligence, speed, and large-area coverage. Regarding research on defects in bolt systems and full-length non-destructive testing, Wu [8], Carlos [9], and Narayanan [10] discovered through testing of bent pipes that modal transformation also occurs in the curved sections of the pipe when excitation is applied in the form of longitudinal waves. Consequently, damage identification becomes extremely challenging under conditions of multimode aliasing and strong noise. Xu et al. [11] employed acoustic emission technology to identify tensile damage patterns in conventional bolts. Lin et al. [12] conducted numerical simulations of stress wave signals in rock masses, then employed the impact-echo method to calculate rock bolt length and grout integrity. Saleem et al. [13] adopted two non-destructive testing methods—impact hammer and ultrasonic acceleration—to identify defective bolts in concrete.

Yuan et al. [14] collected acoustic emission signals from test beams with varying degrees of damage. They fed the waveform data into a convolutional neural network to predict the damage levels of the test beams, thereby validating the effectiveness of the proposed method. Yang et al. [15] employed three distinct types of convolutional neural networks—classification-based, regression-based, and pixel-level image segmentation—for structural damage prediction and identification. They conducted a comparative analysis of the three approaches, evaluating their strengths and weaknesses from perspectives including structural style, computational process, training methodology, and damage prediction accuracy. Sun et al. [16] constructed a defect database using data augmentation methods for identifying plate defect types. Subsequently, they investigated the defect classification performance of Dense Networks, Convolutional Neural Networks, Recurrent Neural Networks, and the newly proposed Deep GFresNet. Hu et al. [17] proposed a method based on multi-scale kernel convolutional neural networks for processing magnetostrictive echo signals. Furthermore, the classification and identification capabilities of convolutional neural network (CNN) models within the scope of image and video processing are also quite impressive. Zhang et al. [18] investigated the application of CNNs in identifying modulation types of digital modulated signals. They acquired and transformed the received baseband data samples of modulated signals to generate constellation-like training images for the convolutional network. Wang et al. [19] addressed the shortcomings of manual evaluation by implementing anchor grout rate classification for measured acoustic reflection signals using an AlexNet convolutional neural network, thereby achieving automated detection of anchor grout density grades. Liu et al. [20] combined wavelet analysis with artificial neural networks to perform wavelet analysis on the dynamic response waveforms of bolt end faces and extract feature vectors. These feature vectors were then input into the network for training, validating the feasibility of the combined wavelet analysis and artificial neural network method for detecting bolt quality. Additionally, neural networks can compensate for human limitations by extracting and classifying features of bolts at different shear angles and positions, enabling efficient identification of micro-scale shear deformation patterns in NPR bolts.

## 3. Modeling of Micro-NPR Bolt Anchoring System

### 3.1. Development of a Shear Model for the Micro-NPR Bolt Anchoring System

**Figure 2:**
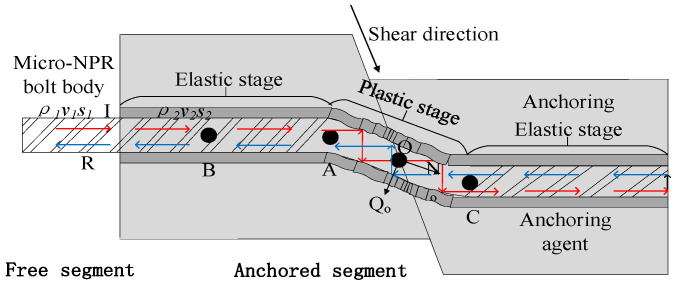
Analysis model of bolt shear “S shape” deformation.

In actual slope support engineering, bolts often undergo deformation under tensile and shear loads. Pellet and Egger [21] developed an “S”-deformation analysis model for rock bolts subjected to tensile and shear loads, facilitating the study of shear behavior in rock bolts, as illustrated in Figure 2.

Point O is the intersection of the bolt and the joint surface. The shear force at point B is zero, while the bending moment at point A is maximum. AO represents the plastic hinge section, with A being the plastic hinge point. N_o_ and Q_o_ denote the axial force and lateral shear force generated at point O during the lateral deformation of the bolt, respectively. The bending moment at point A increases as the plastic zone of the rock expands. When the bending moment reaches its limit, the bolt transitions from the elastic stage to the elastic-plastic stage. At this point, the cross-section of the bolt within the anchorage remains in an elastic state, obeying Hooke’s law. The curvature at the elastic-plastic interface can be regarded as the curvature of an elastic-plastic beam. If the bending moment at Point A exceeds the plastic limit bending moment, two plastic hinges symmetrical to the joint plane will form. It can be assumed that the bolt’s deformation remains unchanged after reaching the plastic hinge and has just begun to rotate. I and R denote the incident and reflected stress waves, respectively. *ρ*_1_, *v*_1_, and *s*_1_ represent the density of the bolt itself, the wave velocity in the free segment, and the cross-sectional area of the bolt body, respectively. *ρ*_2_, *v*_2_, and *s*_2_ denote the density of the anchored section, the wave velocity in the anchored segment, and the cross-sectional area of the anchored section, respectively.

To investigate the application of modular convolutional neural networks in identifying shear deformation of micro-NPR bolts, models were established for micro-NPR bolts after shearing at different angles and positions. Shear angles were set at 0°, 15°, 30°, 45°, 60°, 75°, and 90°. The shear locations were set at 0.75 m, 1.0 m, and 1.25 m from the free end face of the anchor bolt, while maintaining a 0.5 m free segment. These locations were designated as pre-shear, mid-shear, and post-shear sections. The dimensions of the micro-NPR bolt anchoring system are summarized in Table 1.

### 3.2. Simulation Modeling of the Micro-NPR Bolt Anchoring System

Using the finite element software ANSYS 2021, numerical simulations were conducted on micro-NPR bolt of identical length but varying shear angles and positions to investigate the propagation characteristics of stress waves within shear bolts.

The test employed typical micro-NPR bolt dimensions for slope engineering: 18 mm diameter and 1.5 m length. In the simulation, anchorage and grout settings closely followed the dimensions of the laboratory-fabricated anchor test rig, ensuring a 50 cm free anchorage length. The physical parameters for the anchorage system model simulation are specified in Table 2.

Selecting the 8-node solid element satisfies the large deformation requirements of the anchor bolt system after shear failure. Firstly, establish multiple key points. After connecting each pair of key points, generate an anchor bolt cross-section at the coordinate origin. Drag the cross-section along the connecting lines to generate a micro-NPR bolt exhibiting “S-shaped” deformation. Subsequently, establish cylindrical grout and surrounding rock models on both sides of the curved bolt segment. After completing all models, employ the GLUE operation from Boolean operations to merge the entire model. A bonded contact constraint was applied to the interface.

**Figure 3:**
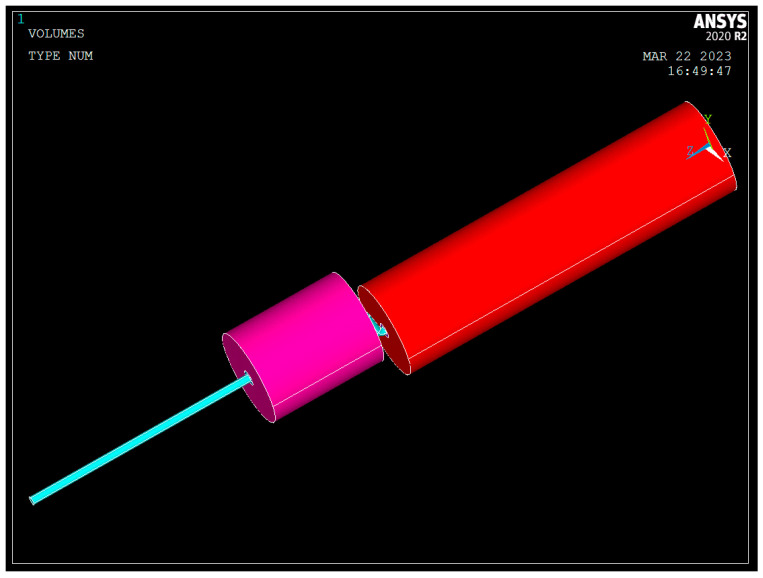
Micro-NPR Bolt Anchoring System Geometric Model.

Apply the Sweep method for meshing the bolt body, grout, and anchorage sections. The geometric model of the micro-NPR bolt anchoring system is shown in Figure 3.

**Figure 4:**
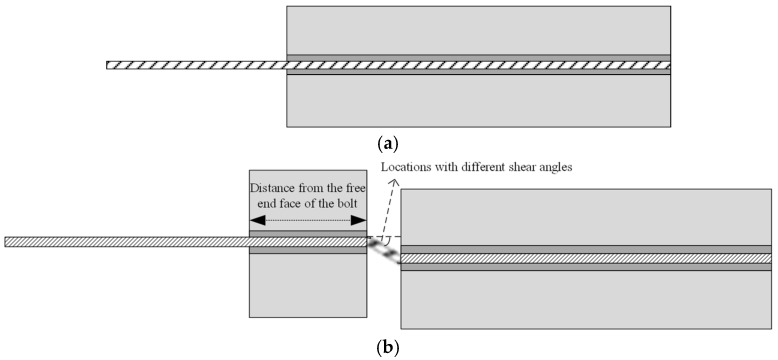
(**a**) Straight bolt; (**b**) Micro-NPR Bolt Anchoring System under Shear.

Shear angles were set at positions 0.75 m, 1.0 m, and 1.25 m from the end face of the 1.5 m-long micro-NPR bolt, with the bend section subjected to 0.05 m of tensile shear. Shear angles ranged from 0° to 90°, incremented every 15°, with seven distinct gradient angles defined. The schematic diagram of the straight bolt is shown in Figure 4a. The schematic diagram of the shear deformation-type micro-NPR bolt anchoring system is shown in Figure 4b.

Stress waves propagating through anchor bolts exhibit severe multimodality and dispersion phenomena, leading to signal waveform distortion and analysis difficulties. Non-destructive testing requires clear, interpretable reflection signals to accurately locate defects and measure lengths. As the excitation signal frequency increases, significant differences appear in the received time-domain waveforms.

The most notable finding is that the received time-domain signal waveform is optimal at an excitation frequency of 20 kHz. As the excitation frequency continues to increase, stress waves exhibit dispersion. Significant dispersion begins at 30 kHz, and as the frequency further increases, dispersion becomes increasingly severe, leading to a substantial reduction in the amplitude of the end-face echo signal.

The Hanning window, a classic window function in signal processing, addresses these challenges of signal attenuation and noise interference with its low spectral sidelobes and energy concentration characteristics. Signals with excessively short periods stimulate numerous unwanted modes and frequency components, exacerbating dispersion. Conversely, overly long periods cause reflected signals to arrive before transmitted signals conclude, resulting in signal overlap that prevents localization. Five periods represent a balanced compromise between these considerations and align with common engineering practices based on guided wave detection theory.

**Figure 5:**
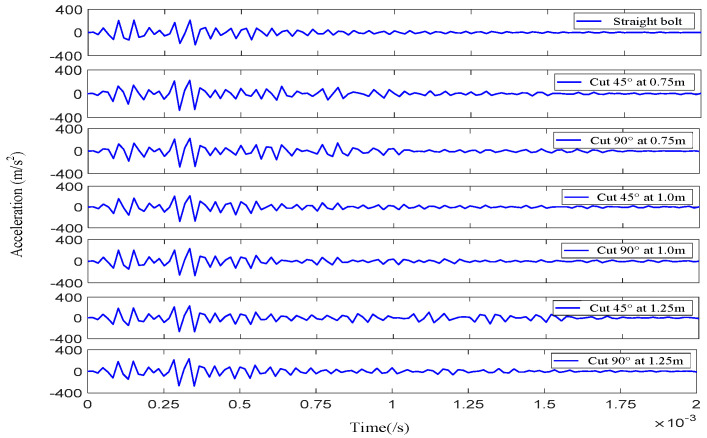
Received waveforms of micro-NPR bolt anchoring system with different shear deformations.

Therefore, a sinusoidal pulse signal modulated through a Hanning window with five cycles was used to excite the free-end face of the anchor bolt. Micro-NPR bolt anchoring system acceleration waveform curves for different shear deformation types were collected, with partial waveforms shown in Figure 5.

### 3.3. Experimental Study on Intelligent Identification of Shear Deformation in Micro-NPR Bolts

**Figure 6:**
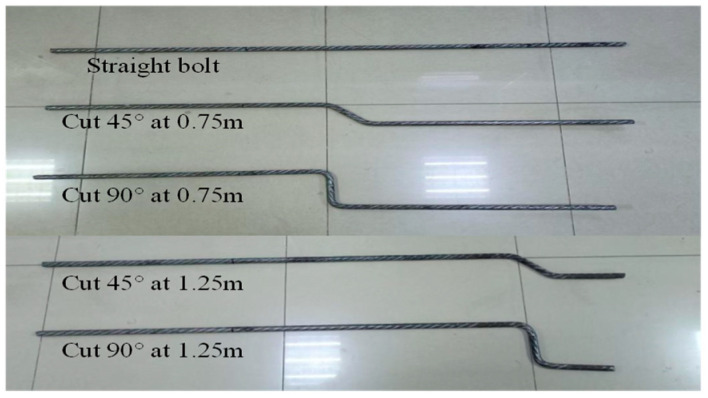
Physical diagram of different shear deformation types of micro-NPR bolt.

This experiment was designed to validate the reliability and accuracy of the preliminary simulation model and further explore the application potential of ultrasonic guided wave technology in practical engineering. To simulate the tensile-shear conditions, micro-NPR bolt shear tests were conducted. The large-scale computer numerical control fully automatic steel bar bending machine enables high-strength and high-toughness bolts, such as the micro-NPR bolt, to undergo an “S”-shaped deformation under shear. Micro-NPR bolts with varying shear angles (0°, 45°, and 90°) and positions (0.75 m, 1.25 m) were categorized as 0, 45F, 45B, 90F, and 90B. The different types of bolts with varying shear deformations are shown in Table 3 and Figure 6.

**Figure 7:**
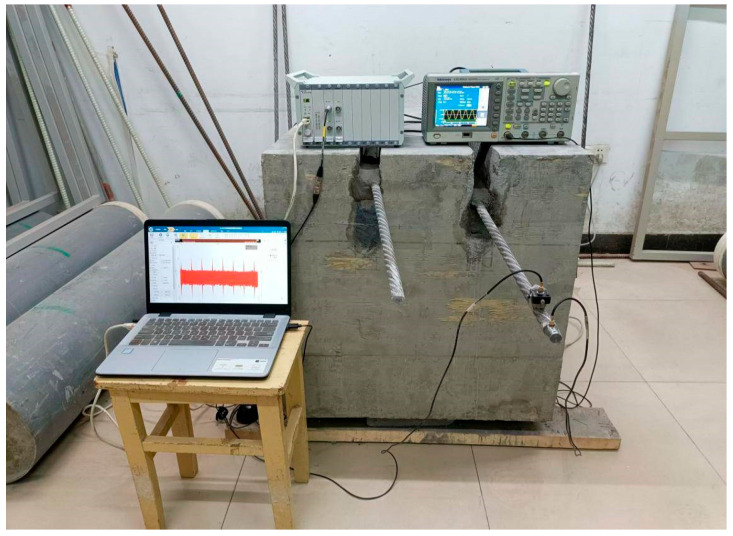
The non-destructive testing signal acquisition experimental platform.

**Figure 8:**
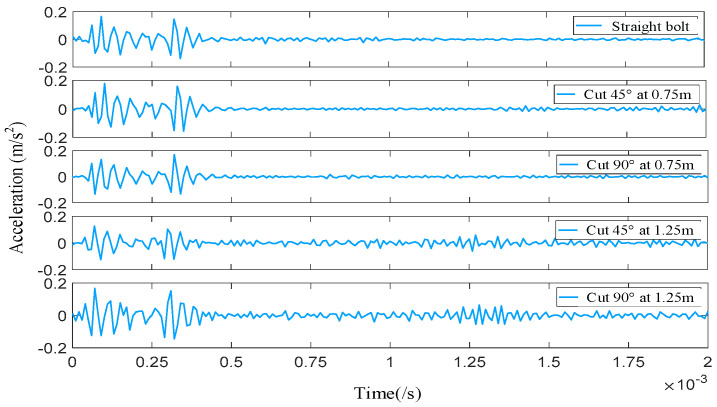
Experimental Data Waveforms from the Bolt Anchoring System with Different Shear Angles and Positions.

The stress wave non-destructive testing experimental platform primarily consists of a signal generator, sensors, a data acquisition instrument, and a host computer. Prior to conducting non-destructive testing experiments, all instrument connections must be verified for accuracy. The signal acquisition process for non-destructive testing of the bolt anchoring system is shown in Figure 7. Acceleration curves for bolt anchoring systems exhibiting different shear deformation types are presented in Figure 8.

It can be observed that the waveform signals obtained from the micro-NPR bolt anchoring experimental platform are extremely similar to those from the simulation model of the same dimensions. However, two differences were identified. In the experiment, the section of the bolt subjected to combined tension and shear elongates under the influence of both tensile and shear forces. However, the simulation cannot perfectly replicate this tensile-shear effect at the bent segment, which may lead to two discrepancies between the experimental and simulated waveforms. The first is that the attenuation occurring in the anchored segment at 0.4–0.6 ms in the experimental data waveform is greater than the attenuation intensity in the simulation. Second, there exists a subtle time difference in the peak values of the wave packets between the experimental and simulated data waveforms in the 1.2–1.4 ms interval. Excluding these two factors, the received waveforms from the simulation model and the experiment remained largely consistent. This further validates the feasibility of simulation-experimental data in intelligent identification methods.

## 4. Design of a Modular Convolutional Neural Network Model

In image processing research, CNNs can extract important features more efficiently and rapidly. The neural network for identifying shear deformation types in micro-NPR bolts, based on a modular CNN architecture, consists of two identification submodules: shear angle and shear location. Different shear angle and position combinations resulting from shearing are fed into separate CNN modules for identification. This approach avoids the low identification accuracy associated with simultaneous identification of both angle and position. The two submodules operate sequentially, outputting identification results for shear angle and position, respectively. The final output combines these results, enhancing classification accuracy. The CNN calculation process is as follows:

(1) Output size of the convolution layer

(1) $$O=\frac{I-k+2P}{S}+1$$

where *O* is out represents the output image size, *I* is in represents the input image size, convolution kernel size is *k*, moving step is represented by *S* and fill number is *P*.

(2) Image output size of the pooling layer

(2) $$O=\frac{I-P_{s}}{S}+1$$

where *P_S_* is the pooling layer size.

(3) Output size of the full connection layer

The output vector length of the fully connected layer was always consistent with the number of neurons.

(4) Number of convolutional layer parameters

Each convolutional layer contains two types of parameters: weight and bias term, and the total number of parameters is the sum of the two types of parameters. Furthermore, the depth of the convolution kernel corresponds to the number of channels of the input image. Therefore, the convolution kernel has *K* × *K* parameters. The number of convolved kernels is indicated in *N*.

(3) $$W_{c}=K^{2}\times C\times N$$

(4) $$B_{c}=N$$

(5) $$P_{c}=W_{c}+B_{c}$$

where *W_c_* is the weight value of the convolutional layer, *B_c_* is the number of bias terms of the convolutional layer, *P_c_* is the number of all parameters, *K* is the kernel size, *N* is the number of cores, and *C* is the number of input image channels.

(5) Number of parameters of the fully connected layer (connected to the last 1 convolution layer)

(6) $$W_{cf}=O^{2}\times N\times F$$

(7) $$B_{cf}=F$$

(8) $$P_{cf}=W_{cf}\times B_{cf}$$

where *W_cf_* represents the number of weights, *B_cf_* is the number of biases, *O* is the size of the output image in the anterior convolutional layer, *N* is the number of nuclei in the anterior convolutional layer, and *F* is the number of neurons in the fully connected layer.

**Figure 9:**
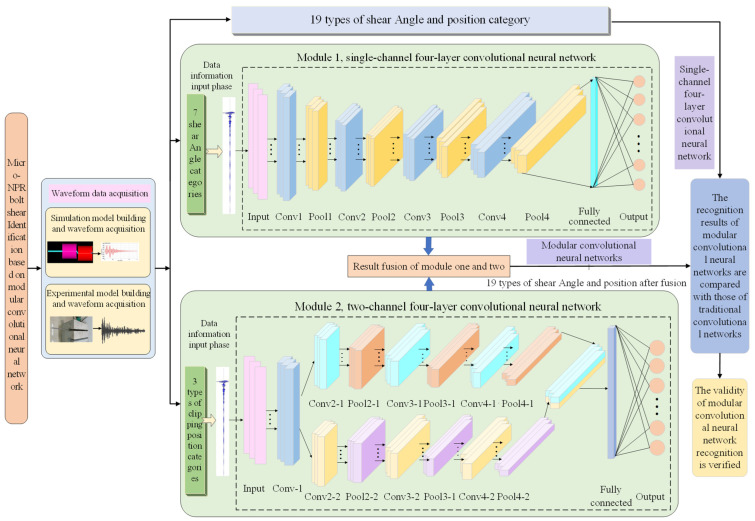
Overall study block diagram.

Due to the limited data volume for various shear angles, a single-channel convolutional network is employed for classification; for the larger data volume of various shear positions, a dual-channel convolutional network is used for classification. Furthermore, by comparing identification results across multi-layer single-channel convolutional network architectures, a four-layer single-channel convolutional neural network with superior performance was ultimately selected. All collected data were allocated in an 8:2 ratio to form the training and test sets, with the training set fed into the convolutional neural network for training. The specific network architecture is shown in Figure 9.

The pseudocode for the dual-module convolutional neural network is shown in Table 4.

## 5. Identification of Shear Deformation Types in Micro-NPR Bolt Anchoring System

### 5.1. Validation and Analysis of Simulation Results for Modular Convolutional Neural Networks

**Figure 10:**
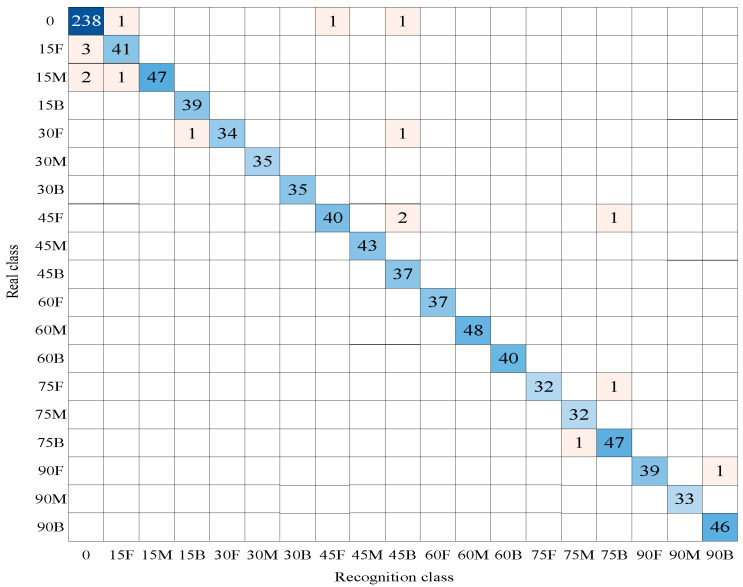
Fusion of Identification Results for Nineteen Shear Angle and Position Categories.

Through non-destructive testing simulations of the micro-NPR bolt anchoring system, data was collected for 2100 sets at different shear angles and positions. Each shear angle type comprised 240 sets. Within the shear position dataset, the straight-bolt anchoring system category contained 240 basic data sets, while other position types each contained 480 basic data sets. The 2100 data sets were randomly shuffled and labeled with different shear deformation tags. After multiple training iterations, the average experimental results for shear angle and position classification are presented in Table 5:

The shear angle sub-module can effectively identify various shear angles of the micro-NPR bolt with an accuracy of 99.479%, while the shear position sub-module can correctly identify the shear position of the bolt with an accuracy of 98.438%. The fusion of identification results from two submodules yielded an excellent outcome with 17 misclassified waveforms and an identification accuracy of 98.229%. The fusion results are shown in Figure 10.

The confusion matrix clearly shows that errors in the dual-module convolutional neural network’s identification of bolt shear deformation angles and positions primarily occur near the diagonal, specifically at adjacent angles or positions. This is because the stress wave patterns corresponding to adjacent angles or positions also tend to be similar, making the identification of adjacent labels a challenging aspect of the task.

Apply Support Vector Machine (SVM), Feedforward Neural Network (FNN), and K-Nearest Neighbors (KNN) algorithm, respectively, for the identification of shear deformation in micro-NPR bolts, and compare the identification results with those obtained from traditional convolutional networks and modular convolutional neural network models. The SVM input signals underwent three-layer wavelet decomposition coefficient analysis. The identification results for each intelligent method are presented in Table 6.

Compared to methods such as SVM, FNN, and KNN that preprocess input signals, the approach in this paper—directly utilizing time-domain signal features for identification —achieved accuracy rates 29.66%, 9.78%, and 8.51% higher, respectively. Compared to traditional CNN approaches, the proposed method—which divides the neural network into shear angle and shear position identification modules—achieves a 7.19% accuracy improvement. This demonstrates that the modular convolutional neural network designed for both shear angle and shear position scenarios of micro-NPR bolts delivers superior identification accuracy.

### 5.2. Validation and Analysis of Experimental Results for Modular Convolutional Neural Networks

**Figure 11:**
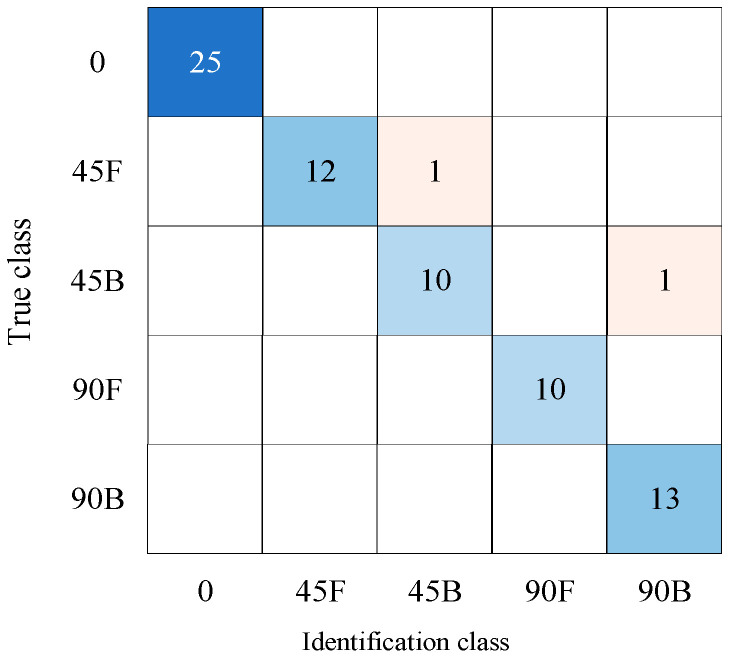
Identification Results for Five Categories of Shear Angles and Positions.

Through non-destructive testing experiments on micro-NPR bolt anchoring systems, data from 360 sets of micro-NPR bolt anchoring systems with varying shear angles and positions were obtained. Straight bolt anchoring system accounted for a significant proportion. The number of basic data sets for straight bolt anchor systems was 120, while other types each had 60 sets. Each waveform contained 200 data points. The 360 data sets, labeled with different shear deformation tags, were randomly shuffled. The experimental results represent the average of multiple training runs. The classification results of the traditional convolutional neural network for shear angle and position showed two misclassified waveforms, achieving an accuracy of 97.22%, as shown in Figure 11 and Table 7.

**Figure 12:**
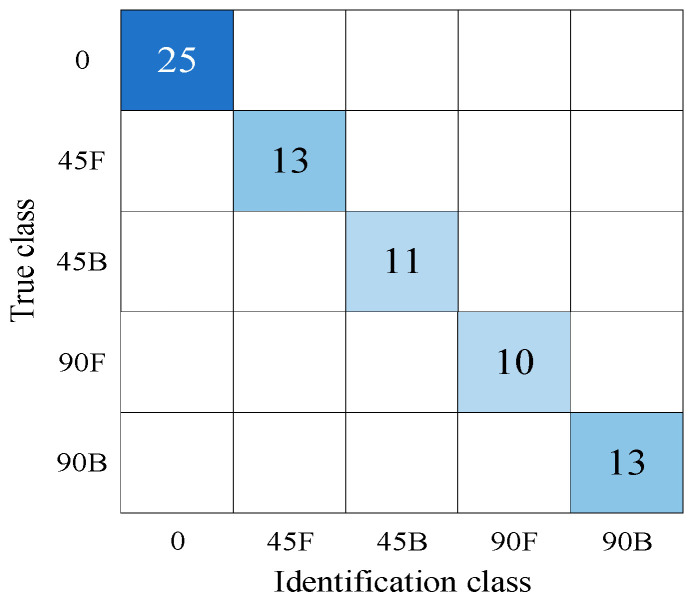
Fusion of identification Results for Five Types of Shear Angles and Locations.

By dividing the neural network into shear angle identification and shear position identification submodules, the results from Module 1 (three shear angle categories) were fused with those from Module 2 (straight bolt and two shear position categories). This fusion achieved zero misclassified waveforms and 100% identification accuracy. Figure 12 shows the confusion matrix for the fusion of identification results across five shear angles and positions.

Table 8 shows the performance of convolutional neural networks in identifying bolt shear deformation, with single modules recognizing angle and position separately, and dual modules performing fusion.

Final validation confirms that the proposed method can effectively identify the shear deformation types of micro-NPR bolts.

We analyzed the waveforms of bolt anchoring systems under different shear deformations and observed a characteristic where the amplitudes of the first two wave packets differ. Additionally, variations in shear angles and other factors result in different elbow echoes and reflections at the front and rear end faces of the elbow section, leading to differences in subsequent waveforms. Although these differences are subtle, they still contribute to achieving high prediction accuracy in convolutional neural network testing. Extending the methodology of this paper to other types of anchor bolts is expected to reveal similar characteristics, which is a direction we aim to pursue in future research.

## 6. Conclusions

This paper proposes a modular convolutional neural network identification method for high-precision monitoring of shear deformation in micro-NPR bolts. By separately processing shear angle and shear position parameters, the method significantly improves identification accuracy and overcomes the limitations of traditional algorithms in multi-parameter joint identification. Experiments demonstrate that the method can effectively support real-time monitoring of bolt deformation in practical engineering, providing a feasible technical approach for timely reinforcement and risk prevention in slope and other related projects, with clear practical application value.

However, the current study also has certain limitations. On the one hand, the model was trained and evaluated solely on a specific type of Micro-NPR bolt. On the other hand, it disregarded numerous complex real-world conditions. In our future work, we will further optimize the model structure, expand its applicability under complex geological conditions, and incorporate all these factors into simulation and experimental setups to more accurately reflect the actual shear deformation behavior of bolts.

## Figures and Tables

**Table 1:** Physical parameters of the 19 bolt anchoring systems with different shear deformations.

Bolt Anchoring Type	Free Segment /m	Cut at 0.75 m			Cut at 1.0 m			Cut at 1.25 m		
		Anchored Segment I/m	Elbow/m	Anchored Segment II/m	Anchored Segment I/m	Elbow/m	Anchored Segment II/m	Anchored Segment I/m	Elbow /m	Anchorage Section II/m
0°	0.5	1.0								
15°	0.5	0.25	0.1	0.75	0.5	0.1	0.5	0.75	0.1	0.25
30°	0.5	0.25	0.1	0.75	0.5	0.1	0.5	0.75	0.1	0.25
45°	0.5	0.25	0.1	0.75	0.5	0.1	0.5	0.75	0.1	0.25
60°	0.5	0.25	0.1	0.75	0.5	0.1	0.5	0.75	0.1	0.25
75°	0.5	0.25	0.1	0.75	0.5	0.1	0.5	0.75	0.1	0.25
90°	0.5	0.25	0.1	0.75	0.5	0.1	0.5	0.75	0.1	0.25

**Table 2:** Physical Parameters of Micro-NPR Anchor Bolt Anchoring System.

Type	Length/m	Diameter/mm	Poisson Ratio	Modulus of Elasticity/GPa	Density kg/m^3^
Micro-NPR bolt	1.5	18	0.01	200	7930
Grout	1.0	32	0.30	210	7800
Surrounding rock	1.0	200	0.20	33	2001

**Table 3:** Physical Parameters for Five Different Shear Deformations of Bolts.

Type of Bolt	Bending Location Distance from Free End/m	Bolt Bending Angle
0	-	0°
45F	0.75	45°
45B	0.75	45°
90F	1.25	90°
90B	1.25	90°

**Table 4:** Convolutional Neural Network Pseudocode.

Modular Convolutional Neural Network Pipeline
**Input:** Input dataset *W*, number of iterations *M*
**Output**: Label data *y* 1. Use the normalization function *f(x)*, *w = f(W)* 2. Initialize the parameters of the 1D-CNN network for the shear angleidentification submodule 3. **for** *m* = 1 … *M* **do**
4. Input the samples for the m-th batch
5. Forward propagation outputs predicted values
6. Calculation error *loss*
7. Update network weights and biases
8. **end**
9. Output the prediction result *y*_1_ from the shear angle identification sub-module
10. Initialize the parameters of the 1D-CNN for the shear location identification sub-module
11. **for** *m* = 1 … *M* **do**
12. Input the samples for the m-th batch
13. Forward propagation outputs predicted values
14. Calculation error *loss*
15. Update network weights and biases
16. **end**
17. Output the prediction result *y*_2_ from the shear location identification sub-module
18. Fuse *y*_1_ and *y*_2_ to obtain y

**Table 5:** Specific values of the identification results of the two sub-modules.

Module	Identification Result Quantity	Training Set	Test Set
Shear Angle identification submodule	Loss indicates the loss function	0.004	0.025
	Identification accuracy	100%	99.479%
	Confusion matrix	-	There are 5 misclassified waveforms
Cut the location identification submodule	Loss indicates the loss function	0.008	0.042
	Identification accuracy	100%	98.438%
	Confusion matrix	-	There are 15 misclassified waveforms
Fusion of identification results	Identification accuracy	100%	98.229%

**Table 6:** Shear Deformation Type Identification for Bolt Anchoring Systems under Different Methods.

Method	Accuracy/%	Mean Squared Error
SVM	68.57	3.293
FNN	88.45	0.694
KNN	89.72	0.568
Traditional CNN	91.04	0.262
Modular CNN	98.23	0.043

**Table 7:** Identification Results for the Five Categories of Shear Angle and Position in Micro NPR Bolts.

	Training Set	Test Set
Loss indicates the loss function	0.0314	0.143
Identification accuracy	97.22%	100%
Confusion matrix	-	Two waveforms were misclassified.

**Table 8:** Identification of Different Shear Angles and Locations in the Micro-NPR Bolt Anchoring System.

Module	Identification Result Quantity	Training Set	Test Set
Shear angle identification submodule	Loss indicates the loss function	0.0429	0.0373
	Identification accuracy	100%	100%
	Confusion matrix	-	All correct
Cut position identification submodule	Loss indicates the loss function	0.0192	0.0542
	Identification accuracy	100%	100%
	Confusion matrix	-	All correct
Fusion of identification results	Identification accuracy	100%	100%

## Data Availability

The data presented in this study are available on request from the corresponding author.

## Abbreviations

The following abbreviations are used in this manuscript:

Micro-NPR	Micro Negative Poisson Ratio
CREA	Constant Resistance Energy Absorption
CNN	Convolutional Neural Network
SVM	Support Vector Machine
FNN	Feedforward Neural Network
KNN	K-Nearest Neighbors
